# Agronomic biofortification in tomato: balancing micronutrient enrichment, yield, and fruit quality

**DOI:** 10.3389/fpls.2026.1835095

**Published:** 2026-05-01

**Authors:** Veneta Stoeva, Viktoria Atanasova, Gergana Marovska, Stanislava Grozeva, Ivanka Tringovska

**Affiliations:** Maritsa Vegetable Crops Research Institute, Agricultural Academy, Plovdiv, Bulgaria

**Keywords:** agronomic biofortification, *Solanum lycopersicum L*., micronutrients, fruit quality, antioxidant capacity, yield components

## Abstract

**Introduction:**

Micronutrient fertilisation is a promising strategy for agronomic biofortification of tomatoes. Yet, its effects on yield formation and fruit quality remain insufficiently clarified, particularly when multiple nutrients and application methods are considered simultaneously.

**Methods:**

This study evaluated a range of fertilisation treatments differing in application method (soil, foliar, and combined) and dose, assessing their effects on fruit mineral composition (Fe, Zn, Mn, Cu), yield components, and quality traits, including physicochemical properties, antioxidant capacity, and an integrated quality score (IQS).

**Results:**

Fertilisation significantly affected fruit mineral composition, with the strongest enrichment observed for Fe, Zn, Mn, and Cu. The highest micronutrient concentrations were recorded at the highest soil application rate (5%), but this treatment caused a substantial yield reduction. In contrast, moderate treatments, particularly 1% soil and 0.1% foliar + 0.5% soil application, achieved significant mineral enrichment while maintaining stable productivity. Yield variation was primarily driven by changes in fruit number rather than fruit weight. Fruit quality traits responded strongly to fertilisation, with higher doses enhancing antioxidant capacity and moderate treatments improving taste balance. The highest IQS was obtained under a combined moderate treatment (0.5% foliar + 1% soil). Principal component analysis revealed a partial trade-off between mineral enrichment and yield, while identifying treatments with balanced performance across mineral composition, productivity, and quality.

**Conclusions:**

The results demonstrate that effective agronomic biofortification of tomato fruits can be achieved through moderate, well-optimised fertilisation strategies. Such approaches enhance micronutrient content without compromising yield or overall fruit quality, providing a practical framework for sustainable improvement of the nutritional value of horticultural crops.

## Introduction

Enhancing the mineral content of edible plant tissues while maintaining stable crop yields remains a significant challenge in horticultural production systems (Szerement et al., 2022). In tomato (*Solanum lycopersicum* L.), the accumulation of mineral elements in fruits is controlled by a complex interaction between soil nutrient availability, plant uptake capacity, transport mechanisms, and sink activity during fruit development. Some essential elements are largely immobile in the phloem (e.g., Ca and Mg), while others (e.g., Fe, Zn, Cu) exhibit moderate phloem mobility (Raiola et al., 2014; Maillard et al., 2015). In practice, all these micronutrients primarily reach the fruit via the transpiration stream. During ripening, the xylem inflow to the fruit often diminishes, limiting mineral import into the fruit (Hocking et al., 2016). As a result, tomato fruits contain a balanced blend of minerals, but their precise composition varies considerably with genotype, environment, and cultivation practices. Although tomato is considered an excellent source of minerals, its nutritional quality can be enhanced only through careful management of cultivar selection, environmental conditions, and agronomic factors such as soil fertility, water management, and salinity (Dorais et al., 2008). Consequently, fruit mineral concentrations frequently exhibit considerable variability across fertilisation regimes, growing systems, and environmental factors, reflecting the physiological constraints on nutrient loading (Avnee et al., 2023).

Agronomic biofortification has been increasingly examined as a strategy to boost the levels of essential micronutrients in edible plant tissues through targeted fertilisation practices (Szerement et al., 2022). In horticultural crops, fertilisation with micronutrients such as Fe, Zn, and Mn has demonstrated potential to enhance fruit mineral composition and nutritional value (White and Broadley, 2009; Çakmak and Kutman, 2018). In tomatoes, both soil and foliar applications have been reported to raise fruit micronutrient concentrations under certain conditions (Rabbi et al., 2024; de Lima Gomes et al., 2025). However, many studies primarily focus on individual elements, while the combined response of multiple micronutrients to fertilisation strategies remains less well understood (Avnee et al., 2023). Because several micronutrients share uptake pathways and transport systems within plants, fertilisation with multi-element formulations may cause coordinated changes in fruit mineral composition rather than independent responses of individual elements (White and Broadley, 2009; Çakmak and Kutman, 2018).

Another significant aspect of agronomic biofortification is its potential interaction with plant growth and yield development. In tomatoes, fruit yield mainly depends on fruit number and size, both of which are strongly affected by reproductive processes and source-sink relationships during plant growth (Kang et al., 2011). Increasing nutrient availability may promote vegetative growth or shift metabolic balance, potentially impacting reproductive development and fruit setting. Moreover, higher mineral concentration in plant tissues may result not only from improved nutrient uptake but also from dilution effects associated with reduced biomass production or altered assimilate distribution (Darch et al., 2022). Differentiating between genuine micronutrient enrichment and apparent concentration variations caused by yield fluctuations remains a key challenge when evaluating fertilisation methods aimed at enhancing the nutritional quality of horticultural crops.

Despite significant interest, clear guidance on how to optimise tomato fertilisation for micronutrient biofortification remains limited. Most studies on tomato have mainly focused on enriching individual micronutrients, especially zinc or selenium. As a result, information on the combined response of multiple mineral elements in tomato fruits under different fertilisation strategies is still scarce. Furthermore, many existing studies explore single application methods or short-term experiments. Also, the comparative effectiveness of foliar, soil, and combined fertilisation approaches for modifying the mineral profile of tomato fruits is still not well understood. Consequently, definitive methodological guidance on how fertilisation strategies can be optimised to improve fruit micronutrient content without harming crop productivity remains lacking.

Therefore, the aim of this study was to evaluate the effects of different microfertilizer application strategies on the mineral composition of tomato fruits by simultaneously analysing several micronutrients and comparing soil, foliar and combined fertilization approaches under multi-year experimental conditions.

## Materials and methods

### Experimental

Experiments were conducted over three consecutive years (2022-2024) in a glasshouse at the Maritsa Vegetable Crops Research Institute, Plovdiv, Bulgaria. The plant material used in the study was tomato (MicroTom). Seeds were sown on April 6 in polystyrene trays with 198 cells filled with a peat–perlite mixture (1:1, v/v). On May 10, seedlings were transplanted into 3-liter containers containing the same substrate, enriched with nutrients to achieve the following approximate nutrient supply per cubic meter: 170 g nitrogen (N), 552 g phosphorus (P^2^O^5^), 250 g potassium (K^2^O), and 32 g magnesium (MgO). These nutrient levels were applied uniformly across all treatments, with the relatively high phosphorus supply reflecting the need to ensure sufficient initial P availability in peat-based substrates, which have low inherent nutrient reserves and limited capacity to sustain long-term nutrient supply. Two plants were grown per pot, and each treatment consisted of three replicate units, with each replicate comprising three pots. For the purposes of statistical analyses, each pot was considered as an experimental unit.

Irrigation was applied uniformly to all treatments using a drip system. Plants were irrigated daily to maintain optimal substrate moisture, avoiding both water deficit and excessive drainage. The irrigation volume was adjusted according to plant growth stage and environmental conditions.

During the experiment, air temperature ranged between 24 and 26 °C, with occasional peaks up to 39 °C, while relative humidity averaged 70-75%. Plants were grown under natural light conditions typical for the May-July growing season in Plovdiv, Bulgaria.

Throughout the entire growing season, plant protection practices were also maintained uniformly across all treatments.

The experiment was conducted using a completely randomized design, with treatments defined as distinct fertilization strategies. with three replicates per treatment. A commercially available micronutrient fertilizer (MiF), containing chelated Cu (0.53%), Fe (3.84%), Mn (2.57%), Zn (0.53%), B (0.52%), Ca (2.57%), and Mo (0.13%), was applied in the study. Eight applications were made during the flowering-to-fruit-set period (approximately early May to late June), with intervals of 3–6 days depending on environmental conditions and slight variations among years, resulting in a total application period of approximately 4–6 weeks. The experimental design comprised three levels of foliar application, three levels of soil application, and four selected combined treatments, representing practically relevant combinations while maintaining a balanced and interpretable set. An untreated control was included for comparison. The following treatments were examined:

1. Control – untreated
2. MiF – foliar application (F) at 0.1% concentration
3. MiF – F at 1% concentration
4. MiF – F at 0.5% concentration
5. MiF – soil application (S) at 0.5% concentration
6. MiF – S at 1% concentration
7. MiF – S at 5% concentration
8. MiF – F at 0.1% concentration + S at 0.5% concentration
9. MiF – F at 0.5% concentration + S at 0.5% concentration
10. MiF – F at 0.1% concentration + S at 1% concentration
11. MiF – F at 0.5% concentration + S at 1% concentration

Different concentration ranges were used for foliar and soil applications to reflect their distinct uptake pathways, with lower concentrations for foliar treatments to prevent phytotoxicity and higher concentrations for soil application due to substrate dilution and root uptake. The selected concentrations represented a gradient from low to high application levels, including a high-dose treatment (5%) to explore the upper response threshold and potential trade-offs between micronutrient enrichment and plant performance. All treatments were applied to a uniform substrate. Peat-based substrates generally contain low and variable amounts of micronutrients, while perlite is inert; therefore, baseline micronutrient levels were low and uniform across treatments, enabling a clear assessment of the microfertilizer effects.

**Figure 1:**
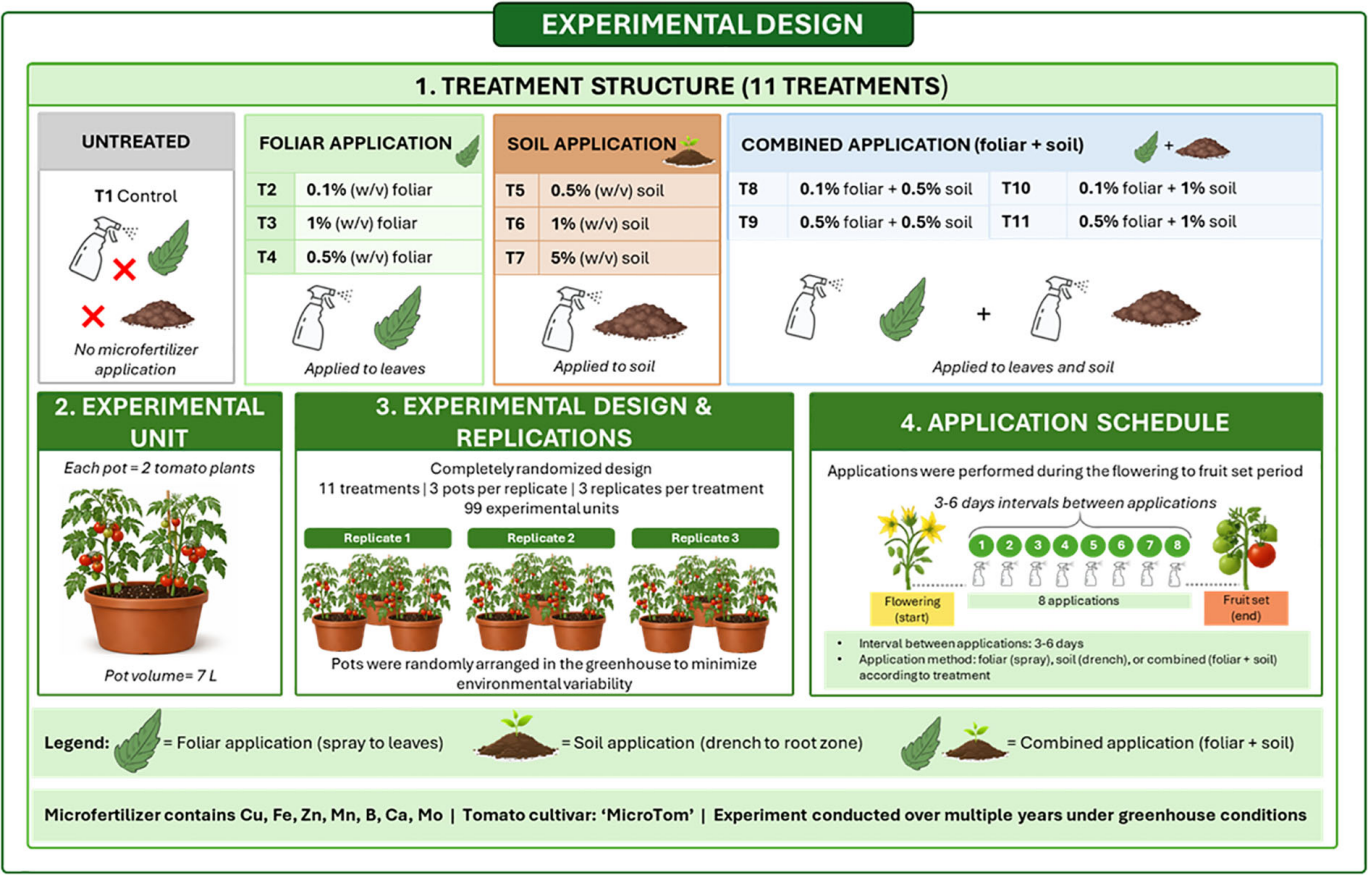
Schematic overview of the experimental design, treatment structure, and application scheme.

A schematic representation of the experimental design and treatment structure is shown in **Figure 1**.

### Measurements and analyses

Yield parameters: total yield per plant, average fruit number per plant, and average fruit weight were recorded at harvest as key indicators of productivity. Measurements were taken from all plants within each replicate. Fruit weight was measured using a precision laboratory balance with 0.01 g accuracy. Further fruit evaluations were performed on fully mature, free of visible defects, fruits.

Fruit firmness and fruit color: measured on three fruits per replicate on two opposite parts in the middle of the longitudinal fruit section. External colour (CIELAB L*, a*, and b* (using chromameter Konica Minolta CR-410, Osaka, Japan) and firmness (using 53215 Fruit Hardness Tester, TR Turoni srl, Forli, Italy) were evaluated.

Fruit sampling: immediately after harvest, fully mature, free of visible defects, fruits from each replicate were pooled, washed, wiped, and divided into halves. Half was freshly homogenised into juice, while the other half was immediately frozen, freeze-dried, and grinded to a fine powder.

Macro- and micronutrient content: elemental analysis was conducted on the dried samples, following microwave-assisted acid digestion in closed Teflon vessels. The concentrations of macro- and micronutrients were quantified using inductively coupled plasma optical emission spectrometry (ICP-OES; Optima 7000 DV, PerkinElmer, USA). Instrument calibration was carried out using certified multi-element standard solutions (CertiPUR^®^, Merck, Germany) to ensure analytical accuracy and traceability. The emission lines were chosen based on previous interference studies. The lines exhibiting low interference and high analytical signal-to-background ratios were selected. The employed emission lines for each mineral were K 766.490, Ca 315.887, Mg 279.077, Na 589.592, B 249.667, Cu 327.393, Fe 259.939, Mn 257.610, Zn 206.200 nm. Physicochemical parameters of fruit quality were analyzed according to standards methods (AOAC International, 2019). Fresh homogenized juice was used for analysis of dry matter content (gravimetrically after oven drying to constant weight, expressed in %), soluble solids content (measured with a digital refractometer and expressed in °Brix); vitamin C content (quantified via titration with 2,6-dichlorophenolindophenol and expressed in mg 100 g^−1^ FW); pH (determined potentiometrically); and titratable acidity, (measured by titration with standardized NaOH and expressed as a % citric acid). Dried material was used for analyses of total polyphenols (mg GAE 100 g^−1^ DW) using Folin-Ciocalteu assay (Singleton and Rossi, 1965), and antioxidant activity (µmol Fe^2+^ g^−1^ DW) by Ferric Reducing Antioxidant Power (FRAP) assay (Benzie and Strain, 1996).

### Data normalisation and quality indexes

To enable the integration of fruit quality traits measured on different scales and units, selected variables were normalised using min–max normalisation. For each variable, normalised values were calculated according to the following equation:


$$X_{norm}=\frac{X_{i}-X_{min}}{X_{max}-X_{min}}$$

where *Xi* is the observed value for a given sample, and *Xmin* and *Xmax* represent the minimum and maximum values of the respective variable across the entire dataset. This transformation rescales all variables to a common range between 0 and 1.

Normalised values were subsequently used to calculate composite quality indices describing different aspects of tomato fruit quality, based on standard mathematical relationships and normalization procedures, enabling integration of variables expressed in different units. All calculated indices are dimensionless.Taste index: fruit taste balance was expressed as a taste index (TI), calculated as the ratio of soluble solids content to titratable acidity


$$TI=\frac{Brix}{TA}$$

Antioxidant capacity index (ACI): calculated as the arithmetic mean of normalized vitamin C content, ferric reducing antioxidant power (FRAP), and total phenolic content (TP)


$$ACI=\frac{VitC_{norm}+FRAP_{norm}+TP_{norm}}{3}$$

Texture score (TS): calculated as the mean of normalized dry matter content and fruit firmness:


$$TS=\frac{DM_{norm}+Firmness_{norm}}{2}$$

Integrated quality score: to provide an integrated evaluation of tomato fruit quality, an Integrated Quality Score (IQS) was calculated as the mean of normalized taste index, antioxidant capacity index, and texture score:


$$IQS=\frac{TI_{norm}+ACI+TS}{3}$$

### Statistics

Statistical analyses were conducted using Jamovi (version 2.6.44.0) and the R statistical environment, version 4.3.3, with RStudio (version 2026.01.1 + 403). Data were tested for normality (Shapiro–Wilk) and homogeneity of variances (Levene’s test), and log-transformed when necessary. Treatment effects were analysed with linear mixed models, considering treatment as a fixed factor and year as a random factor. The significance of fixed effects was assessed using F-tests with Satterthwaite degrees of freedom. Differences between treatments and the control were evaluated through model contrasts derived from the linear mixed model. When a significant treatment effect was observed, pairwise comparisons between treatments were performed using Tukey’s honestly significant difference (HSD) test with p-value adjustments for multiple comparisons. Differences were deemed statistically significant at p < 0.05. Descriptive statistics (means and standard deviations) were calculated from the original, non-transformed data. To aid in interpreting treatment effects relative to the control, the relative change (%) in mineral element concentrations was calculated as:


(Treatment−Control)/Control×100

and visualised as a heatmap.

To examine multivariate patterns in fruit mineral composition across treatments, mean element concentrations were initially calculated for each treatment across all observations. These treatment means were then standardised by element using Z-scores


(z=(x−mean)/SD)

computed separately for each element across treatments. The resulting standardised matrix was visualised as a heatmap with hierarchical clustering applied to both treatments and elements using Euclidean distance. Heatmaps were generated in R using the packages pheatmap, RColorBrewer, and grid.

To further examine multivariate relationships among mineral composition, yield, and fruit quality traits, principal component analysis (PCA) was performed. The analysis included yield, integrated quality score (IQS), and fruit concentrations of Fe, Zn, Mn, and Cu. Before analysis, all variables were standardised using Z-score transformation (mean-centered and scaled to unit variance) to ensure comparability among variables expressed in different units. PCA was carried out on the correlation matrix, and the first two principal components were utilised to visualise relationships among variables and treatments.

## Results

### Mineral composition of tomato fruits under different fertilisation treatments

Mean concentrations of mineral elements in tomato fruits under various fertilisation treatments are presented in **Table 1**. Fertilisation caused the greatest variation in the micronutrients Fe, Cu, Mn, and Zn, while the responses of Ca and Mg were weaker and less consistent across treatments. Among the analysed elements, Fe showed the strongest response, increasing from 35.0 in the control to 137.2 in variant T7. Similar increases were observed for Cu and Mn, which rose from 9.2 to 37.3 and from 21.1 to 49.2, respectively. Zinc also increased across several treatments, reaching 37.7 compared to 21.5 in the control. In contrast, Ca tended to decrease in treatments with strong micronutrient enrichment (from 0.178 in the control to 0.065 under T7), while Mg varied moderately without a clear trend. Overall, the results clearly indicate a treatment-dependent shift in fruit mineral composition, with higher micronutrient levels at increased fertilisation rates. Among all treatments, T7 showed the greatest enrichment. Nutrient concentrations expressed on a fresh-weight basis (**Supplementary Table 1**) showed patterns similar to those observed on a dry-weight basis, indicating that treatment effects were not primarily driven by differences in fruit water content.

**Table 1:** Average concentrations (± SD) of mineral elements in tomato fruits under various fertilization treatments.

Treatment	Micronutrients (mg kg^−1^ DW)					Macronutrients (% DW)			
	B	Cu	Fe	Mn	Zn	Na	Mg	Ca	K
1	11.0 ± 2.7	9.2 ± 1.6	35.0 ± 5.2	21.1 ± 3.2	21.5 ± 2.7	0.10 ± 0.05	0.23 ± 0.07	0.18 ± 0.08	4.54 ± 1.15
2	10.7 ± 3.0	12.1 ± 3.0	49.7 ± 14.9	23.6 ± 3.1	25.5 ± 3.8	0.10 ± 0.04	0.22 ± 0.08	0.16 ± 0.07	4.47 ± 1.13
3	12.2 ± 2.0	19.3 ± 2.3	67.2 ± 16.6	28.2 ± 1.4	31.8 ± 2.1	0.16 ± 0.02	0.30 ± 0.03	0.08 ± 0.02	6.10 ± 0.35
4	15.0 ± 3.3	22.7 ± 5.7	60.5 ± 22.9	35.2 ± 4.7	28.8 ± 4.0	0.13 ± 0.05	0.24 ± 0.06	0.23 ± 0.05	5.29 ± 0.86
5	13.1 ± 2.5	11.2 ± 1.8	38.1 ± 6.5	26.8 ± 2.9	21.6 ± 3.2	0.08 ± 0.03	0.17 ± 0.03	0.20 ± 0.02	3.49 ± 0.78
6	15.3 ± 3.1	14.5 ± 2.8	59.2 ± 29.7	35.5 ± 3.9	29.4 ± 4.2	0.14 ± 0.07	0.22 ± 0.07	0.16 ± 0.06	4.22 ± 1.20
7	25.3 ± 2.7	37.3 ± 28.4	137.2 ± 51.1	49.2 ± 5.1	37.7 ± 3.6	0.28 ± 0.05	0.31 ± 0.02	0.06 ± 0.01	5.84 ± 0.33
8	14.0 ± 1.2	14.3 ± 2.7	58.9 ± 14.4	30.4 ± 6.4	29.7 ± 6.4	0.10 ± 0.04	0.17 ± 0.03	0.17 ± 0.04	3.73 ± 0.45
9	13.5 ± 3.3	15.1 ± 5.7	68.0 ± 22.9	31.0 ± 4.7	29.4 ± 4.0	0.11 ± 0.05	0.19 ± 0.06	0.17 ± 0.05	4.21 ± 0.86
10	18.2 ± 6.4	16.6 ± 3.0	79.1 ± 44.1	36.7 ± 2.7	30.5 ± 4.3	0.19 ± 0.07	0.25 ± 0.07	0.14 ± 0.07	4.77 ± 0.84
11	23.7 ± 6.1	18.9 ± 5.6	64.1 ± 23.8	38.1 ± 13.1	34.2 ± 5.9	0.23 ± 0.10	0.24 ± 0.10	0.12 ± 0.06	4.92 ± 1.50

Values represent mean ± SD (n = 9 experimental replicates; 3 replicates × 3 years).

**Figure 2:**
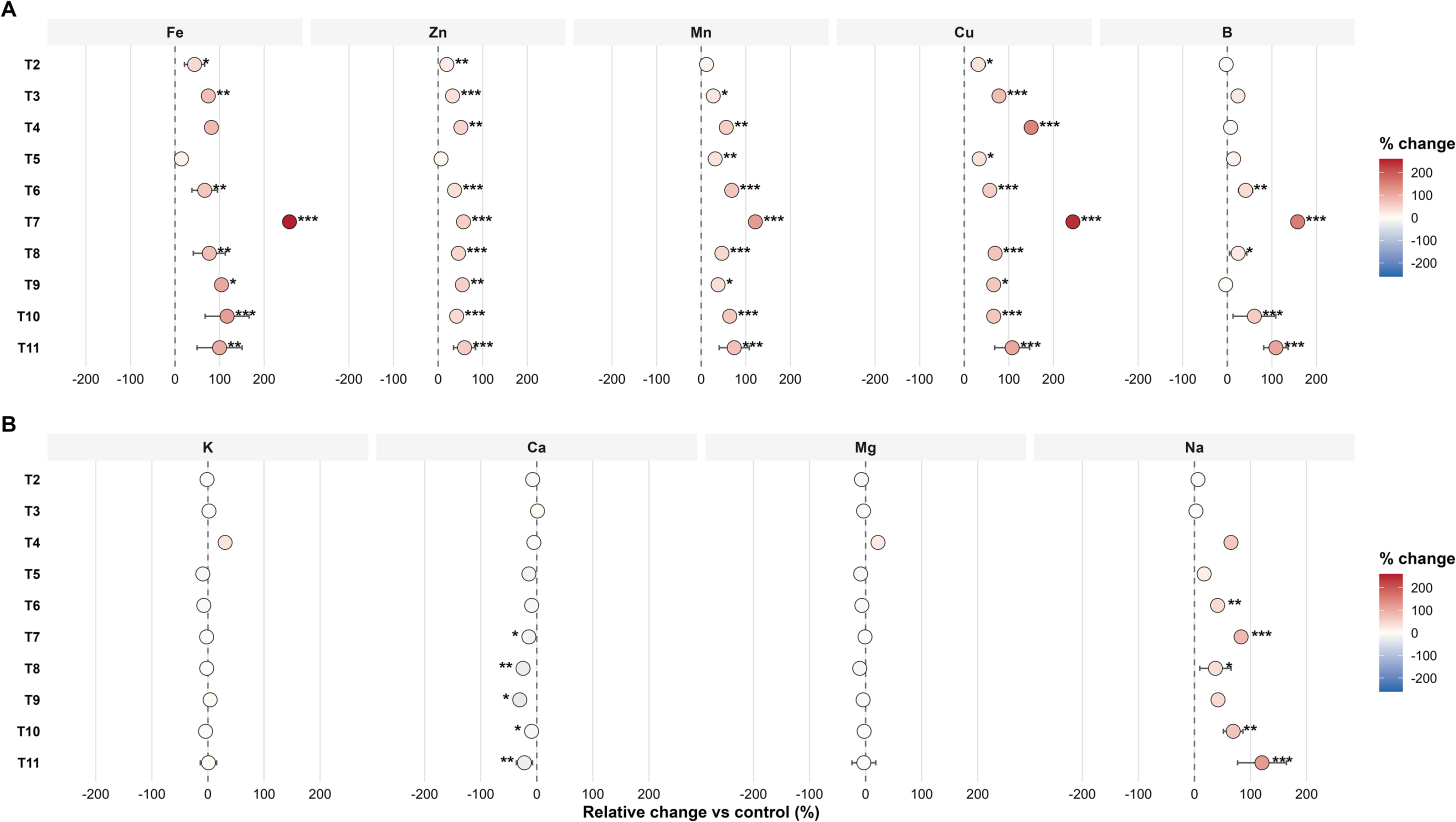
Relative change (%) in mineral element concentrations in tomato fruits under different treatments compared with the control. Values show percentage differences calculated as (Treatment − Control)/Control × 100 using year-specific control means. Circles represent average relative changes across years, and horizontal bars show the standard error. Colour intensity indicates the size of the effect, with red signifying increased and blue signifying decreased concentrations compared to the control. Asterisks mark significant differences from the control (* p < 0.05, ** p < 0.01, *** p < 0.001), based on linear mixed-effects models with treatment as a fixed factor and year as a random factor applied to log-transformed data.

**Figure 2** shows the relative changes in mineral concentrations compared to the control treatment, supporting earlier findings that Fe, Cu, Mn, and Zn exhibited the strongest increases, whereas Ca and Mg showed smaller changes and, in some cases, slight decreases relative to the control. Statistical comparisons based on linear mixed models identified significant differences between some treatments and the control for several elements, as indicated by the significance markers, indicating that the fertilizer mainly affected the micronutrient portion of the fruit’s mineral profile.

**Figure 3:**
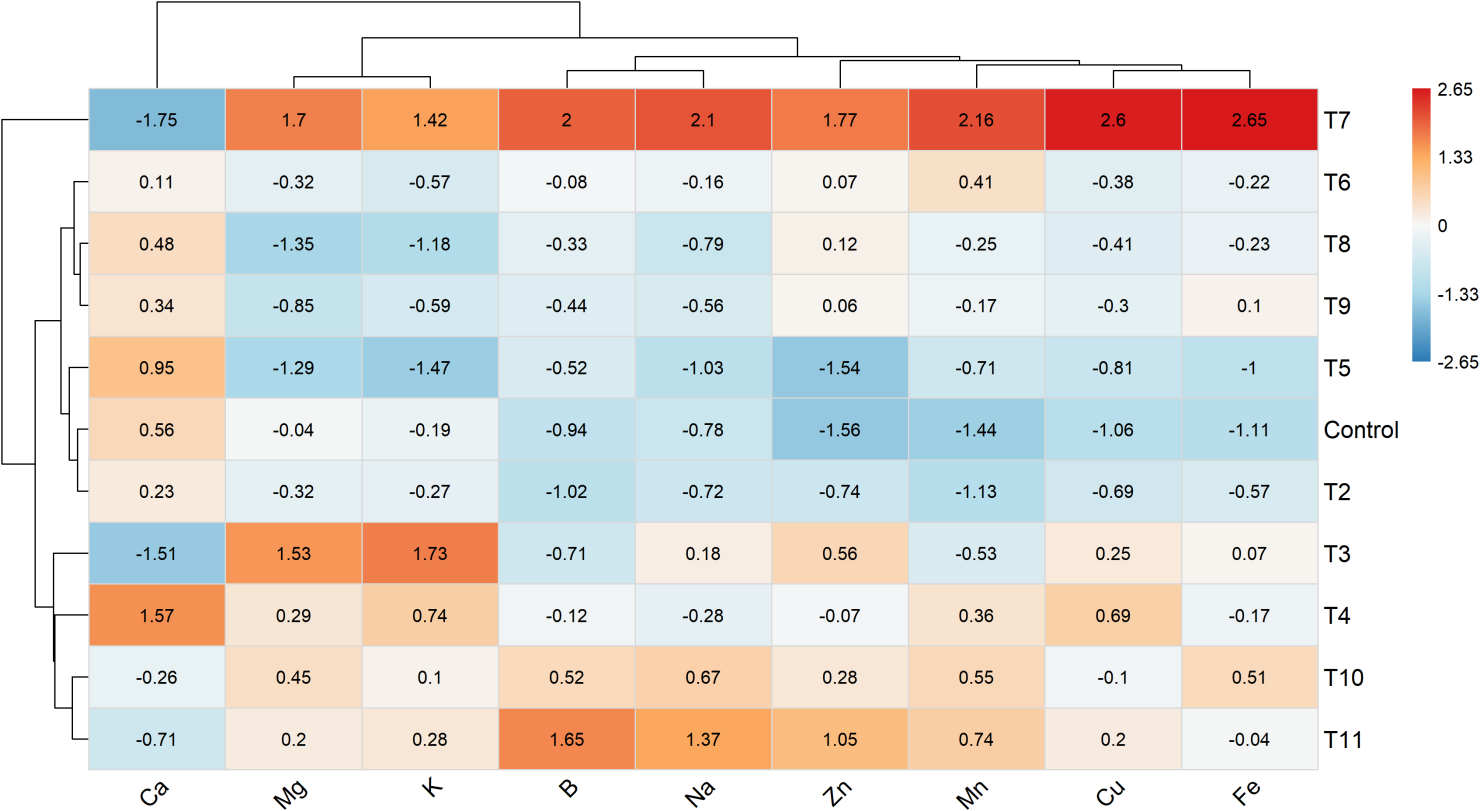
Z-score heatmap with hierarchical clustering illustrating patterns of mineral element concentrations (DW basis) in tomato fruits under different fertilization treatments. Values represent Z-score standardized treatment means calculated separately for each element across treatments. Cell colors indicate deviations from the overall mean for each element, with red representing higher and blue representing lower standardized values. Hierarchical clustering was applied to both treatments and elements using Euclidean distance to identify similarities in mineral composition among treatments and coordinated responses among elements.

The multivariate structure of treatment-level mineral composition is illustrated by the Z-score heatmap with hierarchical clustering (**Figure 3**). This analysis distinguished T7 from the other treatments due to its markedly elevated micronutrient levels. A second group, including T6, T8, and T9, showed moderate increases in several micronutrients, while the control and lower-dose treatments clustered closer to the average mineral profile. Element clustering indicated a coordinated response among Fe, Cu, Mn, and Zn, whereas Ca, Mg, and K formed a separate group with smaller treatment-related changes.

### Yield formation and component responses to treatment

Yield per plant varied greatly among treatments, with several showing significant yield reductions and others exceeding the control level (**Table 2**). The highest yields were observed in the soil-applied MiF at 0.5% (T5) followed by the combined 0.1% foliar + 0.5% soil treatment (T8), both of which surpassed the control. Moderate yields were recorded in T2 and T6, whereas some treatments, especially T7, T9, and T11, resulted in notable yield reductions, with significantly lower yields than the control.

**Table 2:** Yield per plant and yield components of tomato under different treatments.

Treatment No | description		Yield per plant (g)	Average fruit weight (g)	Average fruit number
1	Control	48.4 ± 13.1	4.4 ± 0.4	11.2 ± 3.4
2	0.1% Foliar	54.3 ± 19.5	4.5 ± 0.4	12.2 ± 4.6
3	1% Foliar	16.6 ± 5.8^*^	3.3 ± 0.8^**^	4.9 ± 0.7
4	0.5% Foliar	35.7 ± 10.0^*^	3.8 ± 0.5	9.4 ± 2.5
5	0.5% Soil	66.9 ± 3.5	4.6 ± 0.2	14.5 ± 0.9
6	1% Soil	52.7 ± 20.7	4.1 ± 0.6	13.0 ± 4.7
7	5% Soil	12.0 ± 3.6^**^	3.0 ± 0.4^***^	4.0 ± 1.0
8	0.1% F + 0.5% S	65.4 ± 5.3	4.0 ± 0.4	16.4 ± 1.3^**^
9	0.5% F + 0.5% S	8.0 ± 4.9^***^	2.5 ± 0.6^***^	3.1 ± 1.8^***^
10	0.1% F + 1% S	39.3 ± 21.4	3.9 ± 0.3	10.0 ± 5.1
11	0.5% F + 1% S	4.8 ± 2.4^***^	2.3 ± 1.0^***^	2.0 ± 1.1^***^

Values are presented as mean ± SD. Asterisks indicate significant differences from the control (*p < 0.05, **p < 0.01, ***p < 0.001).

Analysis of yield components showed that the main factor affecting total yield was differences in fruit number rather than fruit weight. The average fruit weight (AFW) stayed fairly consistent across treatments, with only a few exhibiting significantly lower values than the control. In contrast, the average fruit number (AFN) exhibited a broader range of variation and closely followed the overall yield trend. Treatments with the highest yields, especially T8 and T5, had higher fruit numbers, while the lowest-yielding treatments, such as T9 and T11, showed significantly reduced fruit numbers.

**Figure 4:**
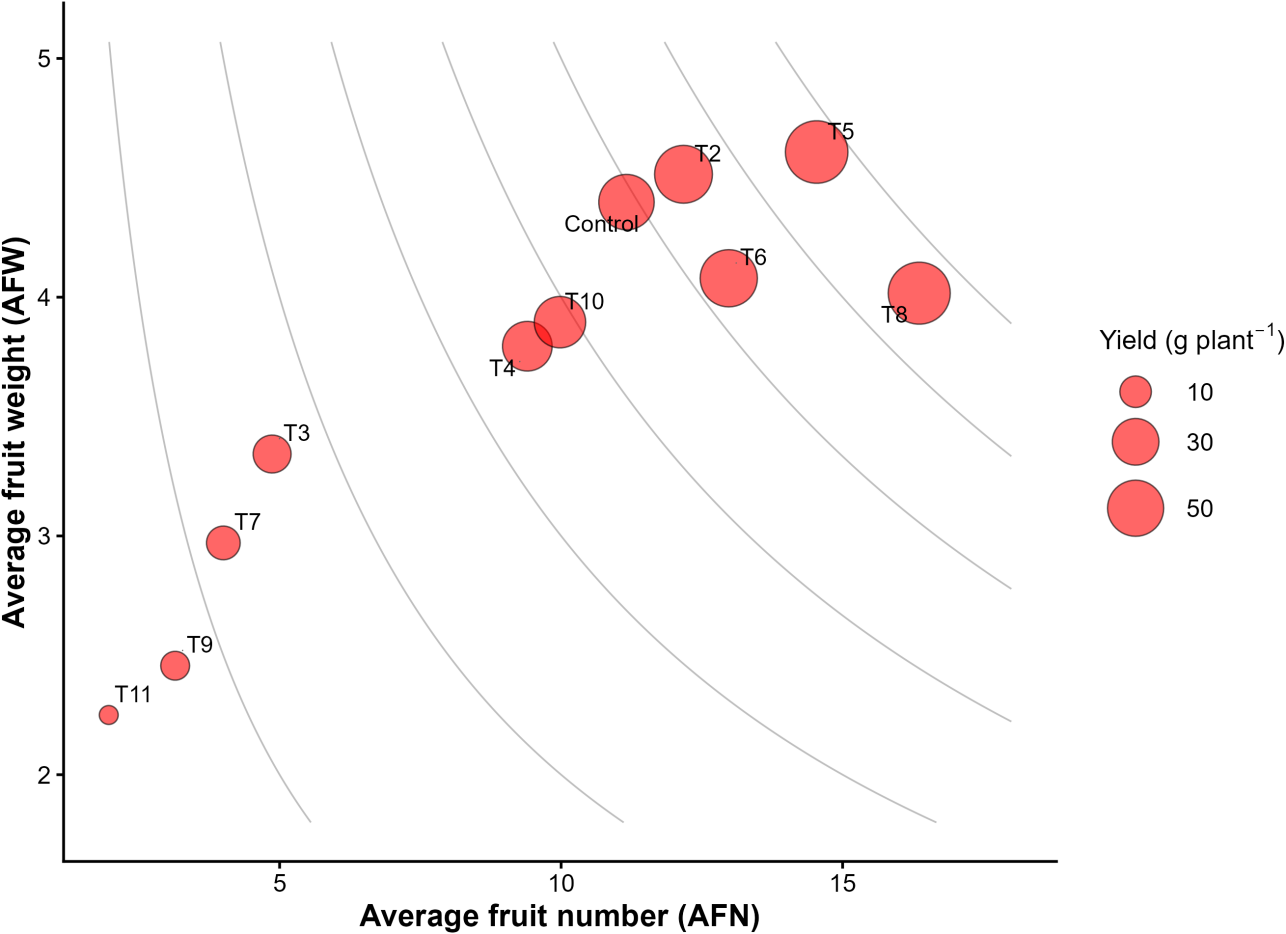
Relationship between average fruit number (AFN) and average fruit weight (AFW) across treatments in tomato. Each point represents the mean value for a treatment (T1–T11). Bubble size represents yield per plant. Grey contour lines represent combinations of fruit number and fruit weight associated with similar yield levels.

The relationship between AFN and AFW across treatments is illustrated in **Figure 4**, where bubble size represents yield per plant. The distribution of treatments along the AFN axis indicates that, as mentioned above, variation in yield was primarily associated with differences in fruit number rather than fruit weight.

**Figure 5:**
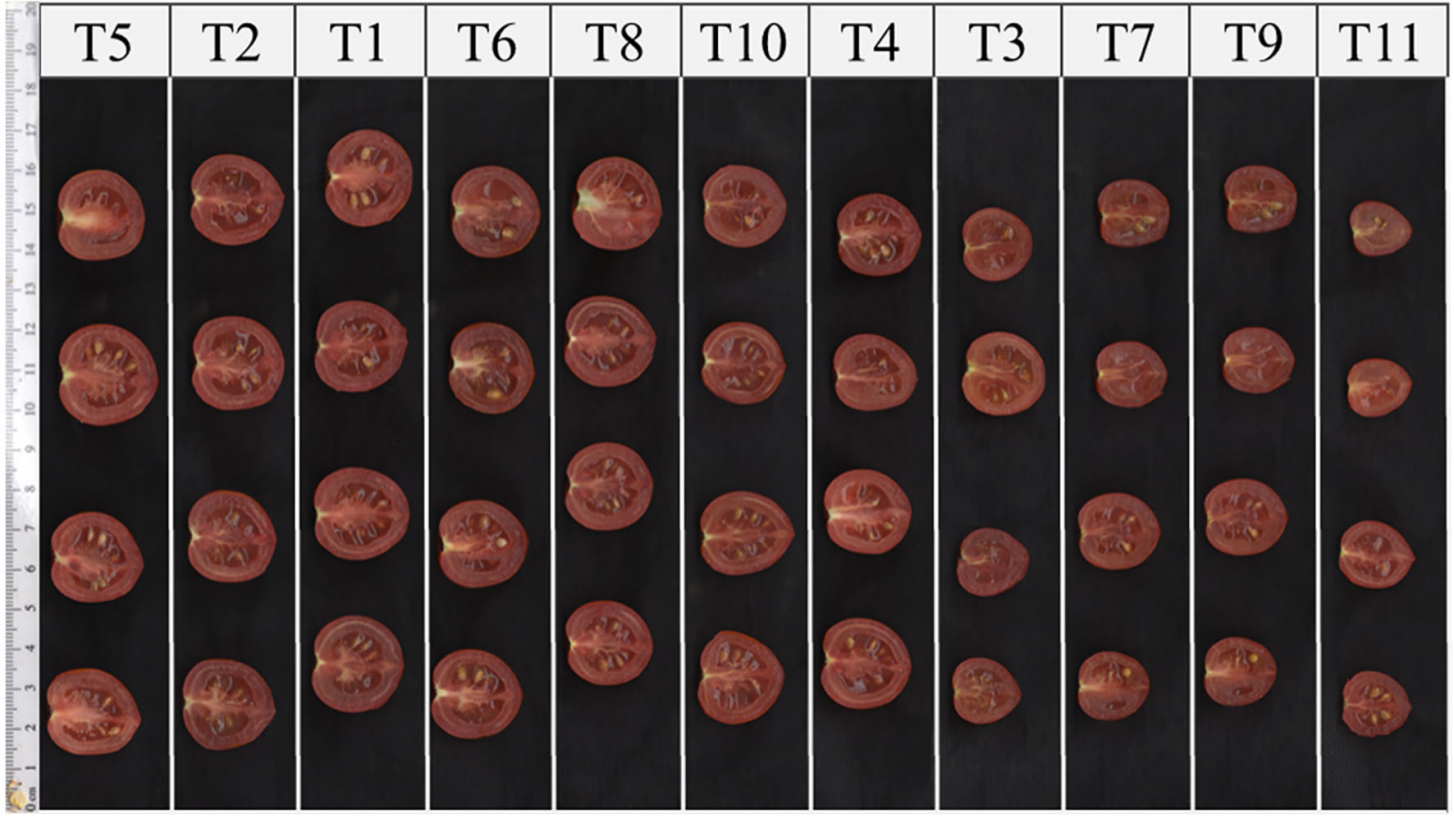
Representative cross-sections of tomato fruits from different treatments (T1–T11), illustrating variation in fruit size and internal structure.

High-yielding treatments (T5 and T8), were located in the area characterized by higher fruit counts combined with relatively consistent fruit weight. Conversely, treatments with the lowest yields (T9 and T11) were found in the lower-left part of the diagram, indicating both fewer fruits and smaller fruit sizes. Most treatments fell within an intermediate zone in which yield differences were mainly associated with variations in fruit number, while fruit weight remained within a relatively narrow range. Representative cross-sections of tomato fruits from different treatments are shown in **Figure 5**, illustrating the variation in fruit size and internal structure associated with the applied fertilization regimes. The visual differences are consistent with the quantitative variation observed in average fruit weight (**Table 2**).

### Treatment-induced variation in physicochemical, antioxidant, and colour traits of tomato fruits

Descriptive statistics for the main physicochemical, antioxidant, and colour traits of tomato fruits are shown in **Table 3**. The fertilisation treatments applied caused distinct differences in their quality profiles, highlighting strong treatment-dependent effects on both primary and secondary fruit metabolism. Marked differences were observed in key compositional traits. Higher dry matter and soluble solids content were recorded in T7 (5% soil) and T11 (0.5% foliar + 1% soil), whereas T4 (0.5% foliar) consistently showed lower values.

**Table 3:** Descriptive statistics of tomato fruit physicochemical, antioxidant and colour traits across treatments (mean ± SD).

Treatment	Dry matter, %	TSS, °Brix	Titratable acidity, % citric acid	Vitamin C, mg 100g^-1^ FW	Firmness, °Shore	pH	FRAP, µmFe^2+^ g^-1^ DW	Total polyphenols, mg eq GAE 100g^-1^ DW	CieLab L*	CieLab a*	CieLab b*
1	8.1 ± 0.4	6.0 ± 0.4	0.53 ± 0.06	31.3 ± 1.9	34.4 ± 3.3	3.99 ± 0.08	75.0 ± 17.9	571.0 ± 138.0	41.3 ± 2.8	27.9 ± 4.9	28.3 ± 5.0
2	8.6 ± 1.2	6.5 ± 1.1	0.52 ± 0.06	32.3 ± 3.6	34.5 ± 4.5	4.00 ± 0.11	82.5 ± 10.9	678.0 ± 178.0	40.6 ± 2.3	26.8 ± 4.5	27.6 ± 4.3
3	9.0 ± 0.6	6.5 ± 0.4	0.64 ± 0.01	45.5 ± 21.6	40.9 ± 3.6	3.87 ± 0.02	93.2 ± 12.8	990.0 ± 317.0	42.8 ± 1.0	31.9 ± 0.4	31.2 ± 2.2
4	6.3 ± 0.9	4.4 ± 0.9	0.54 ± 0.06	25.9 ± 7.6	35.2 ± 4.4	3.99 ± 0.08	91.6 ± 13.6	696.0 ± 168.2	44.4 ± 2.2	32.2 ± 3.1	34.0 ± 3.6
5	9.2 ± 1.5	6.9 ± 1.3	0.49 ± 0.07	32.3 ± 3.1	32.5 ± 4.8	4.04 ± 0.12	86.1 ± 15.6	507.0 ± 58.0	40.0 ± 2.0	25.9 ± 2.6	26.0 ± 3.2
6	8.9 ± 1.5	7.1 ± 1.4	0.55 ± 0.04	31.3 ± 3.2	35.3 ± 2.3	3.97 ± 0.05	82.8 ± 13.6	654.0 ± 278.0	42.0 ± 2.3	30.1 ± 3.0	29.6 ± 3.9
7	10.4 ± 0.8	7.1 ± 1.0	0.76 ± 0.01	45.2 ± 21.9	43.4 ± 2.5	3.91 ± 0.03	91.1 ± 6.5	722.0 ± 121.0	42.6 ± 0.1	35.3 ± 0.5	30.9 ± 0.9
8	8.3 ± 0.8	6.2 ± 0.8	0.48 ± 0.03	30.4 ± 2.0	34.1 ± 6.0	4.04 ± 0.10	71.9 ± 18.8	565.0 ± 109.0	41.6 ± 4.7	25.4 ± 3.5	28.5 ± 7.1
9	9.0 ± 0.9	6.8 ± 0.9	n.d ± n.d	n.d ± n.d	32.1 ± 4.4	3.94 ± 0.08	86.0 ± 13.6	435.0 ± 168.2	39.8 ± 2.2	26.2 ± 3.1	26.6 ± 3.6
10	9.0 ± 0.2	6.5 ± 0.5	0.60 ± 0.09	33.4 ± 2.0	34.1 ± 3.4	3.91 ± 0.05	87.9 ± 6.8	691.0 ± 161.0	42.6 ± 2.2	30.7 ± 3.2	30.5 ± 3.4
11	10.9 ± 0.9	8.0 ± 1.3	0.62 ± 0.19	33.9 ± 8.8	35.4 ± 9.0	3.97 ± 0.14	109.0 ± 19.4	728.0 ± 154.0	43.2 ± 2.1	30.2 ± 5.1	31.2 ± 2.6

n.d. – not determined.

Acidity-related traits also treatments. Titratable acidity was notably higher in T7 (5% soil), while treatments such as T5 (0.5% soil) and T8 (0.1% foliar + 0.5% soil) showed comparatively lower acidity. Despite these variations, fruit pH remained relatively constant, indicating that changes in acidity were primarily due to titratable acid content rather than fluctuations in active acidity.

Antioxidant-related parameters showed pronounced treatment effects. The highest vitamin C and total phenolic content were observed in T3 (1% foliar) and T7, accompanied by increased FRAP, indicating strong activation of antioxidant metabolism. In contrast, T8 exhibited comparatively lower antioxidant capacity, highlighting a weaker metabolic response under this treatment.

Fruit firmness followed patterns similar to dry matter accumulation. The highest values were recorded in T7, followed by T3 and T11, indicating improved structural integrity. Conversely, treatments like T4 and T9 were characterised by reduced firmness.

Colour attributes further differentiated treatments. The most intense red coloration (a*) was observed in T7, suggesting enhanced lycopene accumulation, whereas T4 exhibited higher lightness (L*) and yellowness (b*), indicating less advanced pigmentation.

To provide a comprehensive evaluation of fruit quality, normalized indices representing taste balance, antioxidant capacity, and texture were calculated and combined (**Table 4**). Clear treatment-dependent differences were observed across all indices. The most favourable taste balance was associated with T5, followed by T11, T8, and T6, indicating that both soil and combined fertilization strategies can enhance the sugar-acid balance. In contrast, T4 exhibited the lowest taste index, reflecting its reduced soluble solids content.

**Table 4:** Descriptive statistics of integrated tomato fruit quality indices across treatments (mean ± SD).

Treatment	Taste index (TI)	Antioxidant capacity index (ACI)	Texture score (TS)	Integrated quality score (IQS)
1	11.4 ± 1.6	0.25 ± 0.13	0.42 ± 0.09	0.32 ± 0.04
2	12.7 ± 2.9	0.32 ± 0.11	0.47 ± 0.08	0.35 ± 0.03
3	10.1 ± 0.7	0.49 ± 0.18	0.64 ± 0.12	0.42 ± 0.11
4	8.2 ± 1.3	0.32 ± 0.09	0.27 ± 0.21	0.20 ± 0.04
5	14.8 ± 4.5	0.28 ± 0.06	0.48 ± 0.17	0.42 ± 0.15
6	12.9 ± 3.4	0.31 ± 0.15	0.51 ± 0.14	0.41 ± 0.11
7	9.3 ± 1.3	0.47 ± 0.10	0.82 ± 0.10	0.46 ± 0.08
8	13.1 ± 1.9	0.23 ± 0.12	0.44 ± 0.12	0.36 ± 0.05
9	n.d ± n.d	n.d ± n.d	0.45 ± 0.14	n.d ± n.d
10	10.4 ± 0.4	0.36 ± 0.08	0.50 ± 0.10	0.38 ± 0.07
11	13.4 ± 2.1	0.44 ± 0.10	0.70 ± 0.32	0.56 ± 0.00

n.d. – not determined.

The antioxidant capacity index (ACI) clearly distinguished T3 and T7 as the most responsive treatments, confirming their strong antioxidant profiles. Moderate ACI values were observed in T10 and T11, while T8 showed the lowest antioxidant potential.

The texture score was highest in T7, followed by T11 and T3, reflecting their combined high dry matter and firmness. Lower values were recorded in T4, indicating weaker structural characteristics.

The integrated quality score (IQS) provided a clear ranking of treatments. The highest overall quality was achieved in T11, indicating a balanced improvement across all components. Treatments T7, T3, T5, and T6 also exhibited relatively high IQS values, although each was characterized by a different contribution of individual components. In contrast, T4 showed the lowest IQS, indicating consistently inferior performance.

### Integrated assessment of tomato fruit quality

**Figure 6:**
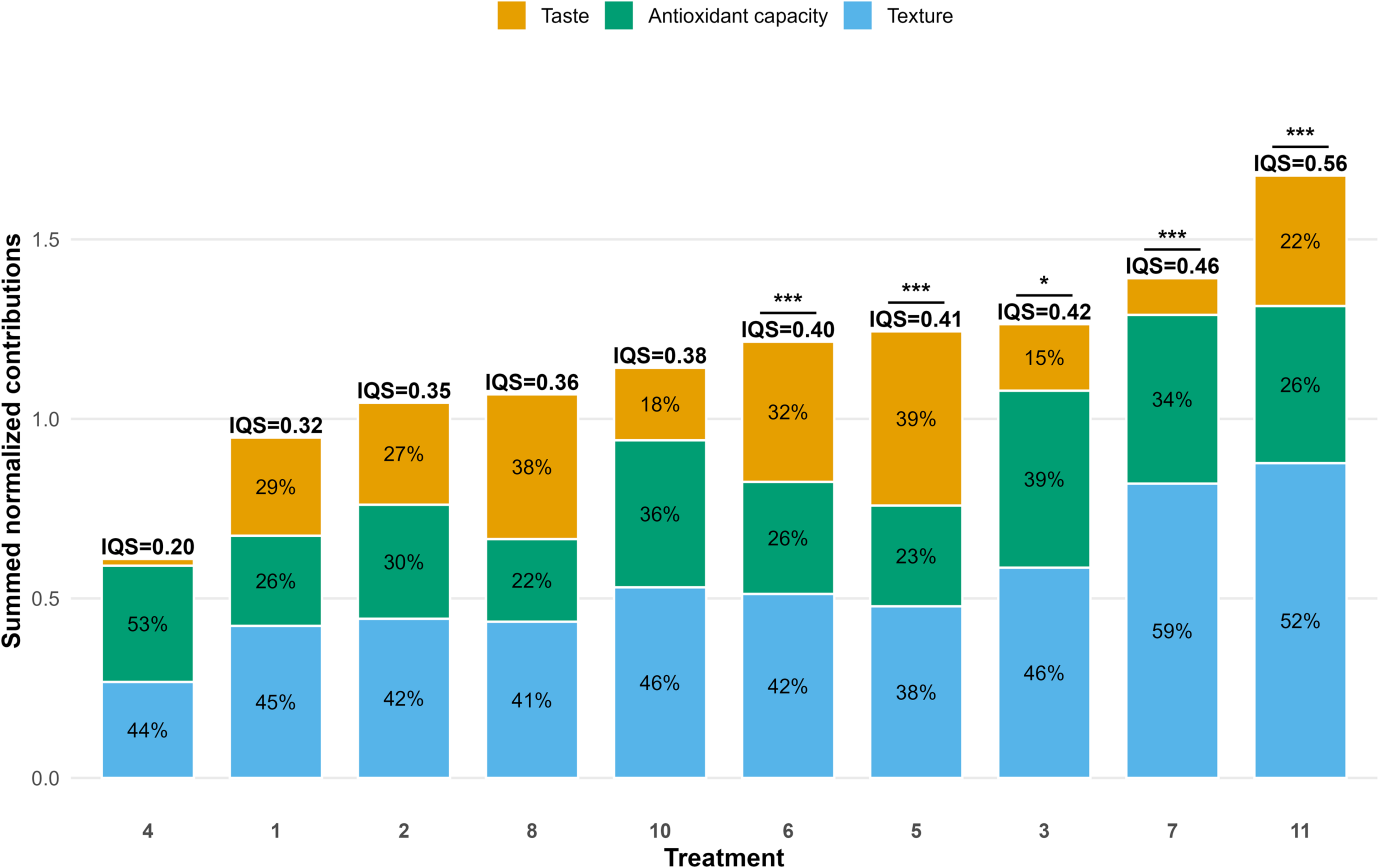
Stacked contributions of the normalized taste index, antioxidant capacity index, and texture score to the integrated quality profile of tomato fruits across treatments. Total bar height shows the combined normalized contribution of the three components. Values above the bars indicate the integrated quality score (IQS). Horizontal lines and asterisks mark treatments that are significantly different from the control (Treatment 1) based on linear mixed-effects model contrasts (* p < 0.05, *** p < 0.001).

To provide a comprehensive evaluation of fruit quality across treatments, normalized indices for taste, antioxidant capacity, and texture were combined into a single quality profile (**Figure 6**). The contribution of each component to the overall normalized quality score varied among treatments.

The integrated quality score (IQS) ranged from 0.20 to 0.56, indicating significant variation in overall fruit quality across the tested treatments. The lowest IQS was observed in T4, while the highest was recorded in T11. Several treatments had intermediate IQS values where the three components contributed more equally to the overall quality profile. In contrast, treatments with higher IQS values displayed a stronger dominance of specific components. For example, T5 showed a higher contribution of the taste component, whereas T3 demonstrated a relatively stronger contribution of antioxidant capacity. In T7 and T11, texture was the dominant component of the integrated quality profile.

Statistical comparisons using linear mixed models showed that several treatments significantly boosted the integrated quality score compared to the control. Specifically, treatments 3, 5, 6, 7, and 11 had significantly higher IQS values, with the greatest improvement seen in treatment 11 (p < 0.001).

The integrated analysis revealed clear treatment-dependent differences in the relative importance of taste, antioxidant capacity, and texture in assessing tomato fruit quality.

### Multivariate analysis of yield, fruit quality, and mineral composition

**Figure 7:**
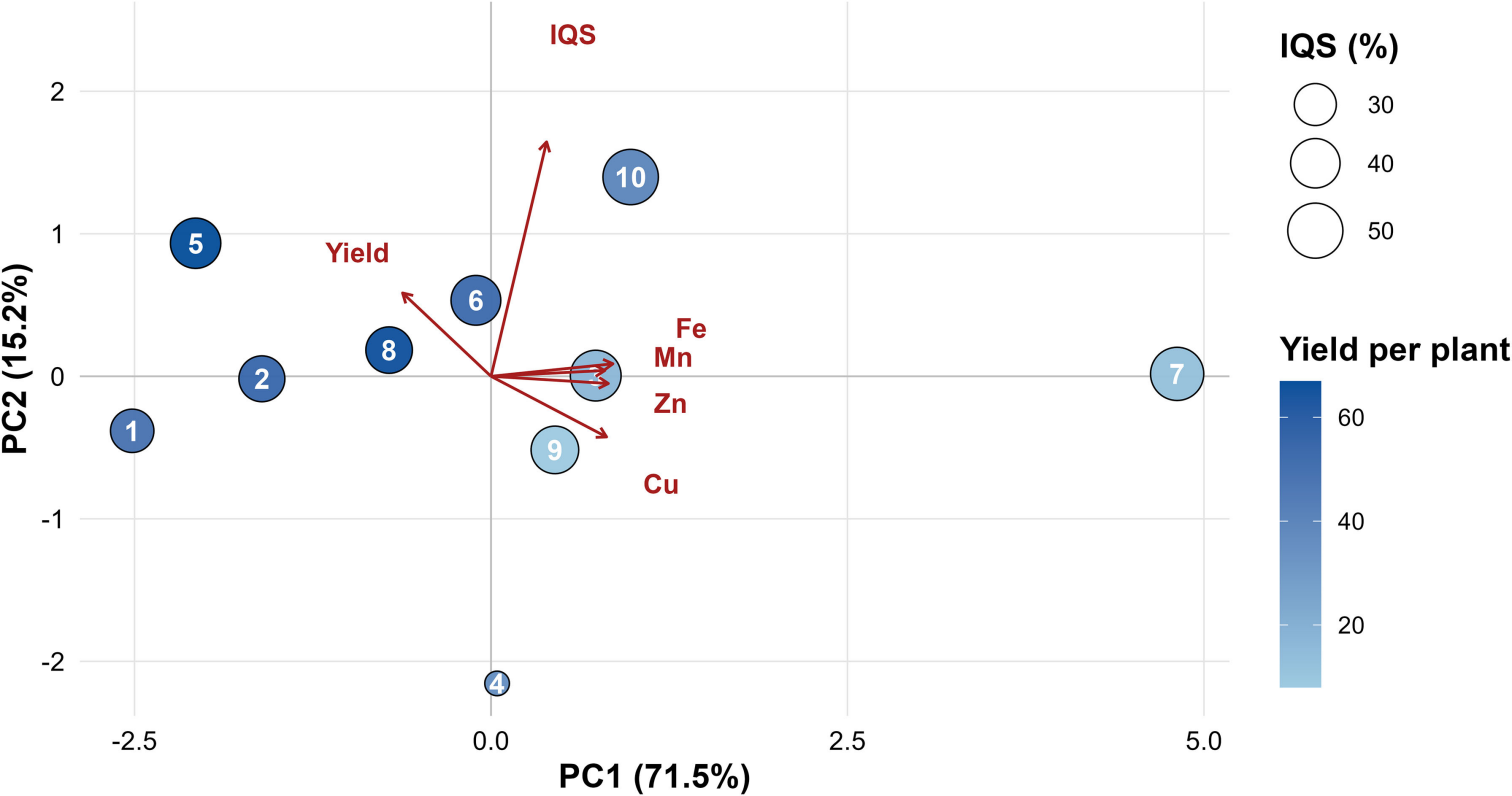
Principal component analysis (PCA) biplot showing the relationships among yield, integrated fruit quality score (IQS), and mineral composition (Fe, Zn, Mn, and Cu) of tomato fruits across treatments. The first two principal components account for 71.5% (PC1) and 15.2% (PC2) of the total variance. Variables were standardised (Z-scores) prior to analysis. Vector directions indicate each variable’s contribution to the ordination. Bubble size reflects the integrated quality score (IQS), while color intensity corresponds to yield per plant.

Principal component analysis (PCA) was conducted to examine the relationships among yield, integrated fruit quality (IQS), and mineral composition (Fe, Zn, Mn, and Cu) across treatments (**Figure 7**). The first two principal components captured most of the variability in the dataset, with PC1 explaining 71.5% and PC2 for 15.2% of the total variance (86.7% combined).

The orientation of the variable vectors showed a strong positive relationship among Fe, Zn, and Mn, which were closely aligned along the positive side of PC1, indicating coordinated variation of these elements across treatments. Copper was also positively associated with PC1 but was partially separated along PC2, suggesting a somewhat independent contribution to the mineral profile.

In contrast, the yield vector pointed toward the negative side of PC1, indicating an inverse association between yield and mineral content within the dataset. Treatments located on the positive side of PC1 were were associated with higher mineral concentrations, while those on the negative side were more closely associated with higher yield values. The IQS vector was mainly aligned along PC2, indicating a pattern largely independent of both yield and mineral composition.

The distribution of treatments in the PCA space revealed several distinct response patterns. Treatments targeting the positive PC1 area were associated with higher mineral concentrations but generally produced lower yields, whereas treatments targeting the negative PC1 area were mainly associated with higher yields and relatively lower mineral levels.

Several treatments occupied intermediate positions between the yield and mineral vectors, indicating a balanced performance that combined mineral enrichment with relatively stable yield and fruit quality. Specifically, treatments T5, T6, T8, and T10 were located in the central PCA region, suggesting a favourable balance among productivity, mineral composition, and overall fruit quality.

Overall, the PCA highlights a partial decoupling between mineral enrichment and productivity, while also revealing treatment-specific differences in the balance among yield, fruit quality, and mineral composition.

## Discussion

The current study examined how different treatments with micronutrients containing fertilizer affect mineral enrichment, yield, and fruit quality in tomatoes. The results showed notable treatment-dependent variations in mineral uptake, productivity, and quality traits, emphasizing the complex interactions between plant nutrition, fruit development, and quality formation.

### Mineral enrichment responses

The present study demonstrates that micronutrient fertilization can significantly modify the mineral composition of tomato fruits, although the extent of the response largely depends on the applied treatment. Among the analysed elements, Fe, Zn, and Mn showed the most consistent enrichment patterns, indicating a coordinated regulation of these transition metals rather than independent element-specific responses. The PCA results further supported this interpretation by revealing a strong positive relationship among these elements, indicating a common “trace-metal enrichment axis”. Such coordination is biologically reasonable because the uptake, transport, and homeostasis of essential transition metals are regulated through interconnected physiological networks with considerable crosstalk between Fe, Zn, and Mn metabolism (Hanikenne et al., 2021; Rai et al., 2021). The observed enrichment patterns align with the physiological functions of these elements in plant metabolism. Fe, Zn, Mg, and Cu serve as essential enzyme cofactors in photosynthesis, respiration, and the regulation of oxidative stress. As a result, variations in micronutrient availability can affect both metabolic activity and nutrient distribution within the plant, although the levels of these redox-active elements must stay within narrow physiological ranges to prevent toxicity (Assunção et al., 2022).

The enrichment observed in tomato fruits also aligns well with the composition of the fertiliser used in this study, which supplied Fe, Mn, Zn and Cu in chelated forms. Chelation stabilises micronutrient ions in solution and reduces precipitation or immobilisation in the soil, thereby prolonging their availability for plant uptake and translocation (Dimkpa and Bindraban, 2016; Klem-Marciniak et al., 2021). Additionally, the timing of nutrient supply may have contributed to the observed responses. Early fruit development in tomato is characterised by an intense but relatively short period of cell division immediately after fruit set, followed by sustained cell expansion. These developmental stages determine sink establishment and can increase the responsiveness of developing fruits to changes in nutrient availability (Fernández and Brown, 2013).

The method of fertiliser application also influences the level of micronutrient accumulation in the tomato fruits. Soil, foliar, and combined applications exhibit distinct enrichment patterns, reflecting differences in nutrient uptake processes. Soil fertilisation mainly supplies nutrients through root absorption and xylem transport, while foliar application enables direct uptake via leaf tissues and can temporarily increase nutrient availability during periods of high metabolic activity. Previous studies have reported similar rises in fruit micronutrient concentrations following foliar fertilisation in tomatoes, particularly for Zn and Fe (Ahmed et al., 2023; Cannata et al., 2025). Combining soil and foliar fertilisation may further enhance nutrient use efficiency by providing complementary uptake pathways and improving overall micronutrient availability to growing fruits (Behera et al., 2025).

The coordinated enrichment of Fe, Zn, and Mn observed in this study is consistent with the known substrate overlap among several metal transporter families. For instance, members of the ZIP transporter family can transport multiple divalent metal ions, offering a mechanistic explanation for the simultaneous increases in these elements when external availability rises (Guerinot, 2000). In contrast, Cu exhibited slightly different multivariate behaviour. Although Cu is essential for many enzymatic processes, but is tightly regulated because excess Cu can cause oxidative damage and disrupt protein function. This strict homeostatic control probably explains the weaker association of Cu with the main Fe–Zn–Mn enrichment gradient observed in the PCA (Yruela, 2009).

Although B was also present in the fertilizer formulation, its behaviour differed from that of the transition metals analysed in this study. Unlike Fe, Zn and Mn, boron transport within plants is strongly linked to transpiration-driven mass flow in the xylem, and its redistribution through the phloem is limited in many species. Consequently, B accumulation in fruits often shows weaker correlations with other micronutrients and may respond differently to fertilization strategies (Brown et al., 2002; Marschner, 2012). This physiological difference may explain why B exhibited a less pronounced response pattern than the transition-metal micronutrients observed in this study.

In contrast to the strong micronutrient response, macronutrients such as Ca (and often Mg) showed smaller, less consistent changes across treatments. In fleshy fruits, Ca accumulation is often limited by xylem transport dynamics and water relations rather than just external supply. Consequently, Ca delivery to fruits is often constrained by internal transport limitations and competition among sinks, thereby reducing the responsiveness of fruit Ca levels to agronomic interventions (Hocking et al., 2016).

To evaluate the nutritional relevance of the observed mineral enrichment, micronutrient levels were also expressed on a fresh-weight basis using the measured fruit dry matter content. Importantly, the relative differences between treatments remained largely consistent after this conversion. The treatments showing higher micronutrient concentrations on a dry-weight basis also retained elevated levels when expressed per fresh weight. In particular, treatment T7 displayed markedly higher concentrations of Fe, Cu, Mn, and Zn compared with the control under both expression bases, confirming that the enrichment represented a real increase in mineral accumulation rather than a simple concentration effect caused by variations in fruit water content. Moderate treatments such as T6, T8, T10, and T11 also maintained higher micronutrient concentrations on a fresh-weight basis, although to a lesser extent than the most intensive treatment. These findings suggest that the treatments mainly promoted mineral uptake and translocation to the fruits rather than simply affecting fruit water relations. Furthermore, the persistence of enrichment after conversion to fresh-weight units emphasises the potential nutritional significance of these treatments for human consumption, as tomatoes are typically eaten fresh.

### Trade-offs between mineral enrichment and yield

A key finding of the multivariate analysis was the partial decoupling between mineral enrichment and yield performance. The PCA clearly showed an inverse association between fruit mineral concentration and yield, with treatments associated with higher micronutrient accumulation generally exhibiting lower productivity. This multivariate pattern reflects a correlation structure between yield and mineral accumulation across treatments rather than a direct causal relationship. This pattern is common in biofortification studies and may indicate dilution effects (Jarrell and Beverly, 1981; White et al., 2009), nutrient antagonism (Xie et al., 2019; Hanikenne and Bouché, 2023), resource allocation trade-offs (Küpper and Andresen, 2016; Krämer, 2024), or stress responses caused by high nutrient availability (Kleiber and Grajek, 2015; Küpper and Andresen, 2016; Kaur and Garg, 2021; Xu et al., 2024). In our study, the persistence of treatment differences after conversion to a fresh-weight basis indicates that the enrichment observed was not solely a concentration effects; rather, the applied micronutrient fertilisation enhanced the overall uptake and accumulation of Fe, Cu, Mn, and Zn in tomato fruits.

The strongest mineral enrichment was observed in treatments associated with reduced yield, particularly in T7, indicating a partial trade-off between micronutrient accumulation and reproductive performance. The relatively low fruit weight and yield observed in these treatments suggest that excessive micronutrient supply may impose physiological constraints on plant growth, affecting both fruit set and fruit development, despite the strong mineral enrichment observed under these treatments. In this context, the highest application rates should be interpreted as experimental boundary conditions rather than practical agronomic recommendations. Such patterns are frequently reported in biofortification studies and may arise from changes in source-sink relationships during fruit development. Tomato yield primarily depends on the combined effects of fruit number and fruit size, with reproductive success and fruit set playing a key role (Heuvelink, 1997). In this study, yield reductions were primarily driven by decreases in fruit number, although reductions in fruit weight also contributed to the overall decline in productivity in several treatments, suggesting that reproductive processes are more sensitive to the treatments than later fruit growth. When the number of fruits declines, assimilates and mineral nutrients are distributed among fewer sinks, resulting in higher concentrations in individual fruits. In addition, the increase in mineral concentrations observed in treatments with reduced fruit size may partially reflect a concentration (dilution) effect, whereby reduced fruit biomass leads to higher measured nutrient levels. This pattern is consistent with the data presented in **Table 1** and **2**, where treatments with smaller fruits generally exhibit higher mineral concentrations, and has been widely reported in crop nutrition studies (Jarrell and Beverly, 1981). However, the persistence of treatment differences after conversion to a fresh-weight basis indicates that the observed enrichment cannot be attributed solely to concentration effects, but also reflects enhanced micronutrient uptake and accumulation. This observations is consistent with studies showing that fruit-set probability is closely linked to the source–sink balance and assimilate availability (Ariizumi et al., 2013; Osorio et al., 2014). Therefore, moderate disturbances in nutrient supply or metabolic balance may primarily influence flower retention and fruit set, ultimately reducing yield (Dorais et al., 2008). A threshold response was also observed, with yield decreasing significantly at application levels above 0.5%. This indicates that while moderate micronutrient supply can support plant performance, higher concentrations may cause physiological constraints that limit both vegetative growth and reproductive development.

Despite the common trade-off between mineral enrichment and yield, several treatments achieved a more balanced performance by combining relatively stable productivity with higher micronutrient levels. From an agronomic biofortification standpoint, treatments such as T6, T8, and T10 appear particularly promising, as they offer an improved mineral profile without significantly reducing crop yield (White and Broadley, 2009).

### Integrated quality responses under micronutrient fertilization

Tomato is recognised as an important dietary source of antioxidant compounds, including ascorbic acid, phenolics, and carotenoids, which enhance the fruit’s overall nutritional value (Ali et al., 2020; Collins et al., 2022). In the current study, in addition to mineral enrichment, the applied micronutrient fertilization induces complex and coordinated changes in tomato fruit quality, reflecting the interplay between primary metabolism (sugars and organic acids), secondary metabolism (antioxidants), and structural development. Variability in fruit quality traits is widely reported in tomatoes across different cultivation systems and nutrient regimes, highlighting the fruit’s metabolic flexibility. In line with previous research, our findings confirm that nutrient management, particularly micronutrient fertilization, plays a key role in shaping tomato nutritional quality through its effects on the accumulation of soluble metabolites and antioxidant compounds (Dorais et al., 2008; Ahmed et al., 2024; Mal et al., 2025; Teshome et al., 2025). Changes in antioxidant levels in response to cultivation practices are often linked to modifications in plant metabolic pathways related to stress responses or altered nutrient availability (Fanasca et al., 2006; Sun et al., 2024). In particular, moderate physiological stress or nutrient-driven metabolic shifts can stimulate the synthesis of antioxidant compounds as part of the plant’s defence system against oxidative stress (Cannea and Padiglia, 2025). The relatively high FRAP values and phenolic content observed in several treatments, therefore, likely reflect these metabolic adaptations.

At the highest applied doses, T3 (1% foliar) and T7 (5% soil) exhibited the greatest antioxidant capacity, indicating a strong activation of secondary metabolic pathways. Such responses are typically linked to moderate physiological stress or changes in nutrient availability, which stimulate the biosynthesis of ascorbic acid and phenolic compounds (Sun et al., 2024; Cannea and Padiglia, 2025). The simultaneous increase in firmness and dry matter in these treatments further supports the idea of a metabolically active and structurally reinforced fruit phenotype. Notably, T7, which combines high antioxidant capacity, improved texture, and intense colouration, though it does not have the highest taste index. This highlights a trade-off among different quality components, where increased accumulation of structural and protective compounds may not coincide with optimal sugar-acid balance. Similar trade-offs have been widely reported in tomato, where shifts in carbon allocation and metabolic priorities affect both nutritional and sensory attributes (Dorais et al., 2008; Quinet et al., 2019; Wu et al., 2023).

In contrast, T5 (0.5% soil) showed the highest taste index but only moderate antioxidant and texture scores, suggesting that sugar and acid accumulation can be partly separated from antioxidant metabolism. This observation agrees with previous studies indicating that flavour-related traits and antioxidant compounds are regulated by overlapping but not identical metabolic pathways (Kaur et al., 2023; Gao et al., 2025; Lu and Zhu, 2022).

The most balanced response was observed in T11 (0.5% foliar + 1% soil), which achieved the highest IQS by combining relatively high values across all quality components. This suggests that the combined foliar and soil application at moderate levels provides optimal conditions for coordinated metabolic activity, enabling simultaneous enhancement of taste, antioxidant capacity, and texture. Similar synergistic effects of combined agronomic practices have been reported in tomato and other horticultural crops (Dorais et al., 2008; Zhang et al., 2025).

Treatments with intermediate responses (T6:1% soil, T8: 0.1% foliar and 0.5% soil and T10: 0.1% foliar and 1% soil) exhibited more stable but less pronounced improvements, reflecting conditions where nutrient supply supports fruit development without strongly stimulating secondary metabolism. In contrast, T4 (0.5% foliar) consistently showed reduced performance across all indices, indicating that this fertilization regime did not effectively support either primary or secondary metabolic processes.

Overall, the results suggest that improving tomato fruit quality through micronutrient fertilisation is not a simple process but relies on maintaining a balance among metabolic pathways. The highest overall quality is not associated with the maximum expression of a single trait, but with the coordinated enhancement of multiple components. In this context, T11, and to a lesser extent T6 and T8, provide a balanced improvement in fruit quality without significant metabolic trade-offs.

### Implications for biofortification strategy and fertiliser management

The present study provides valuable practical insights for developing agronomic biofortification strategies in tomatoes. The results clearly show that maximising micronutrient concentration in fruits does not always produce the best agronomic outcoms. Instead, the success of biofortification relies on finding fertilisation methods that balance nutrient enrichment with crop yield and overall fruit quality.

From a management perspective, moderate fertilisation strategies represent the most efficient and sustainable approach. Treatments such as T6 (1% soil), T8 (0.1% foliar and 0.5% soil), and T10 (0.1% foliar and 1% soil) consistently achieved higher concentrations of Fe, Zn, Mn, and Cu without significant yield penalties, suggesting that biofortification can be successfully applied with relatively low input levels. This is particularly relevant for practical agriculture, where economic efficiency and input optimisation are crucial considerations (Faure et al., 2025). This pattern also supports a broader agronomic conclusion: nutrient management can improve both yield and nutritional quality simultaneously when the nutrient source, rate, timing, and crop context are optimised (Ishfaq et al., 2023).

Foundational biofortification synthesis and Zn-focused agronomic reviews emphasize that success depends heavily on fertilizer form and application strategy, and that methods to improve delivery to edible tissues are key to successful agronomic biofortification (Çakmak and Kutman, 2018; Praharaj et al., 2021; Bhardwaj et al., 2022). Our findings also emphasise the importance of the application method. Combined soil and foliar applications demonstrate a clear advantage in achieving balanced responses, likely due to complementary uptake pathways that improve nutrient availability during critical developmental stages. This indicates that integrated fertilisation strategies may offer greater flexibility and efficiency than single-application methods.

At the same time, the strong negative effects on yield observed at high application rates (e.g., T7) highlight the risks associated with excessive micronutrient supply. These responses emphasise that biofortification strategies should consider not only nutrient availability but also plant physiological limits and potential stress responses. Therefore, establishing optimal dose thresholds is essential to avoid negative impacts on reproductive processes and yield formation.

## Conclusions

This study shows that agronomic biofortification with micronutrients in tomatoes causes coordinated changes in mineral content, yield, and fruit quality, with responses strongly influenced by application rate and method. Significant increases in Fe, Zn, Mn, and Cu were observed across multiple treatments, confirming the effectiveness of micronutrient fertilization as a strategy to enhance the nutritional quality of tomato fruits.

However, the results clearly show a trade-off between mineral enrichment and productivity. The highest enrichment levels, especially under the maximum fertilization treatment (T7), consistently correlated with lower yields, mainly due to limitations in fruit set but also accompanied by decreases in fruit weight in several treatments. This indicates that excessive micronutrient supply may create physiological constraints on reproductive development.

A threshold response was observed, with yield decreasing at higher application levels, emphasizing the narrow margin between beneficial and inhibitory effects of micronutrient inputs. Conversely, moderate and combined application methods (notably T5, T6, and T8) achieved a more favorable balance by keeping yield relatively stable and enhancing mineral composition.

Fruit quality responses further support this pattern, indicating that optimal quality is achieved through balanced modulation of multiple traits rather than maximisation of individual parameters. Multivariate analysis confirmed that improvements in mineral content, yield, and quality are only partially aligned, emphasising the need for integrated evaluation.

Taken together, these findings demonstrate that optimisation is essential in agronomic biofortification, as both insufficient and excessive micronutrient supply may limit overall performance. The present study provides a framework for identifying application thresholds and balanced fertilisation strategies, offering practical guidance for achieving simultaneous improvements in nutritional value, yield, and fruit quality in tomato production systems.

## Data Availability

The original contributions presented in the study are included in the article/Supplementary Material. Further inquiries can be directed to the corresponding author.
