# Supplementary material for: Agronomic biofortification in tomato: balancing micronutrient enrichment, yield, and fruit quality
Source: Front Plant Sci. 2026 May 1;17:1835095. doi: 10.3389/fpls.2026.1835095 (PMC13176317; doi:10.3389/fpls.2026.1835095)
Supplement: Supplementary file 1 [file Table1.docx]

**Table S1.** Micronutrient concentrations in tomato fruits expressed on a fresh-weight basis calculated from measured fruit dry matter content

| Treatment | Micronutrients (mg kg⁻¹ FW) | | | | | Macronutrients (% FW) | | | |
| --- | --- | --- | --- | --- | --- | --- | --- | --- | --- |
|  | B | Cu | Fe | Mn | Zn | Na | Mg | Ca | K |
| 1 | 0.9 ± 0.2 | 0.7 ± 0.1 | 2.9 ± 0.4 | 1.7 ± 0.2 | 1.8 ± 0.2 | 0.008 ± 0.004 | 0.021 ± 0.006 | 0.017 ± 0.005 | 0.367 ± 0.086 |
| 2 | 0.9 ± 0.3 | 1.0 ± 0.2 | 4.3 ± 1.7 | 2.0 ± 0.2 | 2.2 ± 0.5 | 0.008 ± 0.004 | 0.017 ± 0.009 | 0.017 ± 0.005 | 0.380 ± 0.086 |
| 3 | 1.1 ± 0.1 | 1.7 ± 0.3 | 6.0 ± 1.3 | 2.5 ± 0.2 | 2.9 ± 0.3 | 0.013 ± 0.006 | 0.030 ± 0.000 | 0.010 ± 0.000 | 0.547 ± 0.031 |
| 4 | 0.9 ± 0.3 | 1.4 ± 0.6 | 3.8 ± 2.5 | 2.2 ± 0.5 | 1.8 ± 0.5 | 0.010 ± 0.006 | 0.020 ± 0.007 | 0.010 ± 0.003 | 0.330 ± 0.077 |
| 5 | 1.4 ± 0.3 | 1.3 ± 0.3 | 5.2 ± 2.5 | 3.2 ± 0.6 | 2.6 ± 0.5 | 0.012 ± 0.007 | 0.018 ± 0.008 | 0.016 ± 0.005 | 0.372 ± 0.099 |
| 6 | 1.2 ± 0.1 | 1.0 ± 0.1 | 3.5 ± 0.9 | 2.5 ± 0.5 | 2.0 ± 0.6 | 0.008 ± 0.004 | 0.015 ± 0.005 | 0.020 ± 0.000 | 0.313 ± 0.045 |
| 7 | 2.6 ± 0.2 | 4.0 ± 3.3 | 14.5 ± 6.5 | 5.1 ± 0.2 | 3.9 ± 0.3 | 0.027 ± 0.006 | 0.033 ± 0.006 | 0.010 ± 0.000 | 0.607 ± 0.040 |
| 8 | 1.2 ± 0.1 | 1.2 ± 0.1 | 5.0 ± 1.6 | 2.5 ± 0.4 | 2.5 ± 0.7 | 0.008 ± 0.004 | 0.013 ± 0.005 | 0.017 ± 0.005 | 0.312 ± 0.044 |
| 9 | 1.2 ± 0.3 | 1.4 ± 0.6 | 6.1 ± 2.5 | 2.8 ± 0.5 | 2.6 ± 0.5 | 0.010 ± 0.006 | 0.020 ± 0.007 | 0.020 ± 0.003 | 0.380 ± 0.077 |
| 10 | 1.7 ± 0.7 | 1.6 ± 0.3 | 8.4 ± 4.6 | 3.3 ± 0.3 | 3.0 ± 0.3 | 0.018 ± 0.005 | 0.025 ± 0.010 | 0.013 ± 0.005 | 0.470 ± 0.062 |
| 11 | 2.7 ± 1.0 | 2.0 ± 0.8 | 7.6 ± 3.0 | 4.0 ± 1.8 | 3.6 ± 0.9 | 0.023 ± 0.012 | 0.023 ± 0.012 | 0.013 ± 0.006 | 0.493 ± 0.197 |

Values represent **treatment means ± standard deviation** (n = 9 experimental replicates; 3 replicates × 3 years).
Micronutrient concentrations on a fresh-weight basis were calculated using the measured dry matter content of the fruits according to the equation:

FW concentration = DW concentration × (Dry matter / 100).

Dry weight is presented at Table 3.

Statistical analyses were performed on **dry-weight data using linear mixed models (LMMs), with treatment** as a fixed factor and year as a random factor (see Table 1).
